# Facilitators and barriers to clinical practice guideline-consistent supportive care at pediatric oncology institutions: a Children’s Oncology Group study

**DOI:** 10.1186/s43058-021-00200-2

**Published:** 2021-09-16

**Authors:** Aaron J. Sugalski, Tammy Lo, Melissa Beauchemin, Allison C. Grimes, Paula D. Robinson, Alexandra M. Walsh, Nancy Santesso, Ha Dang, Brian T. Fisher, Andrea Rothfus Wrightson, Lolie C. Yu, Lillian Sung, L. Lee Dupuis

**Affiliations:** 1 University of Texas Health Science Center at San Antonio, San Antonio, USA; 2 Department of Research and Evaluation, Kaiser Permanente, Pasadena, USA; 3 Columbia University Irving Medical Center, New York, USA; 4 Pediatric Oncology Group of Ontario, Toronto, Ontario Canada; 5 Center for Cancer and Blood Disorders, Phoenix Children’s Hospital, Phoenix, USA; 6 University of Arizona, Phoenix, USA; 7 Department of Health Research Methods, Evidence and Impact, McMaster University, Hamilton, Canada; 8 Children’s Oncology Group, Monrovia, USA; 9 Department of Population and Public Health Sciences, University of Southern California, Los Angeles, USA; 10 Pediatrics and Epidemiology, The Children’s Hospital of Philadelphia and the Perelman School of Medicine at the University of Pennsylvania, Philadelphia, USA; 11 Clinical Research Nurse Coordinator, Nemours Center for Cancer and Blood Disorders, Wilmington, USA; 12 LSUHSC/Children’s Hospital, New Orleans, USA; 13 Research Institute, The Hospital for Sick Children, 555 University Ave, Toronto, ON M5G 1X8 Canada; 14 Faculty of Medicine, University of Toronto, Toronto, Canada; 15 Leslie Dan Faculty of Pharmacy, University of Toronto, Toronto, Canada

**Keywords:** Pediatrics, Oncology, Clinical practice guideline, Supportive care

## Abstract

**Background:**

Clinical practice guideline (CPG)-consistent care improves patient outcomes, but CPG implementation is poor. Little is known about CPG implementation in pediatric oncology. This study aimed to understand supportive care CPG implementation facilitators and barriers at pediatric oncology National Cancer Institute (NCI) Community Oncology Research Program (NCORP) institutions.

**Methods:**

Healthcare professionals at 26 pediatric, Children's Oncology Group-member, NCORP institutions were invited to participate in face-to-face focus groups. Serial focus groups were held until saturation of ideas was reached. Supportive care CPG implementation facilitators and barriers were solicited using nominal group technique (NGT), and implementation of specific supportive care CPG recommendations was discussed. Notes from each focus group were analyzed using a directed content analysis. The top five themes arising from an analysis of NGT items were identified, first from each focus group and then across all focus groups.

**Results:**

Saturation of ideas was reached after seven focus groups involving 35 participants from 18 institutions. The top five facilitators of CPG implementation identified across all focus groups were organizational factors including charging teams with CPG implementation, individual factors including willingness to standardize care, user needs and values including mentorship, system factors including implementation structure, and implementation strategies including a basis in science. The top five barriers of CPG implementation identified were organizational factors including tolerance for inconsistencies, individual factors including lack of trust, system factors including administrative hurdles, user needs and values including lack of inclusivity, and professional including knowledge gaps.

**Conclusions:**

Healthcare professionals at pediatric NCORP institutions believe that organizational factors are the most important determinants of supportive care CPG implementation. They believe that CPG-consistent supportive care is most likely to be delivered in organizations that prioritize evidence-based care, provide structure and resources to implement CPGs, and eliminate implementation barriers.

**Trial registration:**

ClinicalTrials.gov Identifier: NCT02847130. Date of registration: July 28, 2016.

**Supplementary Information:**

The online version contains supplementary material available at 10.1186/s43058-021-00200-2.

Contributions to the literature

- Although we speculated that pediatric oncology healthcare professionals might approach CPGs differently, their perspectives regarding barriers and facilitators of CPG implementation are similar to those of other healthcare professionals.
- Pediatric oncology healthcare professionals believe that their organization’s ability to cultivate strong relationships between stakeholders and allocate resources to initiate and sustain the changes required by CPG-consistent care is critical to CPG implementation.
- Conversely, they believe that CPG implementation is more difficult when organizations do not value evidence-based practice, care standardization, or collaboration.
- Incomplete acceptance of the importance of supportive care may influence supportive care CPG implementation in pediatric oncology.

## Background

Clinical practice guidelines (CPGs), defined as care recommendations “informed by a systematic review of evidence and an assessment of the benefits and harms,” [1] are the foundation for translating evidence into practice. Delivery of CPG-consistent care has improved patient outcomes in many contexts [2–5]. Supportive care in oncology is commonly defined as the “prevention and management of adverse effects of cancer and its treatment.” [6] Comprised of more than 200 member institutions, the Children’s Oncology Group (COG) is the world’s largest organization focused on pediatric oncology research. Recognizing the potential of supportive care CPGs to optimize care, the COG endorses rigorously developed supportive care CPGs that are consistent with the COG’s mandate [7]. Each COG member institution is encouraged to adopt or adapt the recommendations of endorsed CPGs as their standards of care.

Implementation frameworks have been developed to guide successful CPG implementation. Elements important to successful CPG implementation have been identified including CPG characteristics, accommodations required for implementation and roles of individuals, organizations, and systems [8]. Nevertheless, uptake of CPGs and adherence to them are poor [9–11]. Almost nothing is known about the extent to which CPG-consistent supportive care is provided to pediatric oncology patients or how to improve the use of CPGs in pediatric cancer. For example, a recent systematic metareview of 25 systematic reviews categorized barriers and facilitators of CPG implementation. 960 primary studies conducted in over 20 countries, in varied settings (e.g., primary, secondary, and tertiary care; low, middle-, and high-income countries) and in varied specialties (e.g., cardiology, infectious disease, obstetrics, psychiatry) were included [12]. None of the included systematic reviews was specific to pediatric oncology.

The pediatric oncology community may approach CPGs from a different perspective from others. For example, pediatric oncology clinical research is highly integrated with care [13] and wide-spread adherence to cancer treatment and standard of care protocols in pediatrics may lead clinicians to under-appreciate the need to consider the strength of a supportive care CPG recommendation before implementation. Conversely, the perception of children as vulnerable and the heavy reliance on standardized treatment protocols to direct care may lead to hesitation in applying approaches that are not explicitly directed by treatment protocols. Thus, pediatric health care professionals may be prone to dismissing strong CPG recommendations or accepting conditional recommendations without adaptation to their context.

Importantly, purposeful identification of facilitators and barriers of CPG uptake is recommended before designing CPG implementation interventions [14, 15]. As a step toward developing supportive care CPG implementation strategies specific to our community, we undertook this study to understand facilitators and barriers of supportive care CPG implementation at pediatric oncology institutions. We focused on supportive care CPGs in general and, more specifically, on CPGs addressing fever and neutropenia management (FN), chemotherapy-induced nausea and vomiting prevention (CINV), and fertility preservation (FP).

## Methods

This qualitative, COG study (ACCL15N1CD; NCT02847130) was approved by the National Cancer Institute (NCI) Pediatric Central Institutional Review Board. The need for documentation of consent was waived; participants provided verbal consent. This analysis is one of three sub-studies within the primary protocol.

All 37 COG-member, NCI Community Oncology Research Program (NCORP) institutions were invited to open ACCL15N1CD and 26 chose to participate in the study. The NCORP is a federally funded network of American institutions that supports cancer research in community settings [16]. Supportive care and cancer care delivery are two research focus areas of the NCORP.

We chose to achieve our study aims through focus groups rather than surveys or one-on-one interviews to approximate the dynamic interprofessional and interdisciplinary collaboration that ideally occurs in CPG implementation. Through this process, we hoped to facilitate a rich discussion. We used the Standards for Reporting Qualitative Research to direct this report [17].

### Participants

Healthcare professionals, excluding trainees, who provided direct care to pediatric oncology patients (e.g., physicians, nurses, pharmacists, social workers, dieticians, psychologists, child life specialists, and physiotherapists) at COG-member, NCORP institutions were eligible to participate.

Institution personnel sent e-mail invitations to solicit focus group participants. Respondents provided demographic information via electronic survey. Participants were purposively selected to ensure variability by profession, years of experience, and location.

### Focus group procedures

Participants traveled to attend face-to-face focus groups at convenient locations. Acknowledging the potential for power imbalance within interprofessional medical teams, we held three types of focus groups: physician-only, non-physician only, and mixed. Each was comprised of four to eight participants and was moderated by co-investigators (AS, AG, LS, LLD, MB) trained in focus group technique and experienced in nominal group technique (NGT; a consensus-building technique that ensures consideration of all participants’ opinions) [18] and cognitive probing (a technique to elicit deeper thinking and discussion on a topic) [19].

We planned to hold a minimum of four focus groups in total, including at least one of each type (physician only, non-physician only, and mixed) and involving at least 32 participants. Focus groups were to be held until saturation of ideas, defined as the identification of no further substantive, novel ideas or themes during review of the coded concepts generated by the focus groups [20], had been reached. Once the minimum number of focus groups and participants had been reached, saturation was assessed by comparing the ideas and themes generated by the last focus group to those generated by the preceding focus groups.

A focus group moderator guide (Additional file 1) was developed by the study team iteratively using the methods of Krueger [21, 22], the Checklist of Determinants of Practice of Flottorp et al., [14] and the CPG implementability framework of Gagliardi et al. [8] to direct its structure and content. The use of the detailed frameworks of Flottorp et al. [14] and Gagliardi et al. [8] enhanced the moderator’s ability to follow-up and probe appropriately following comments made by focus group members. Study team members piloted the moderator guide among themselves before the first focus group.

Each focus group consisted of two parts. Part 1 started with an orientation to CPG implementation. Participants discussed how CPGs were incorporated into care at their institution, how often CPGs were used in their practice, and how CPGs fit into their daily workflow. Next, the moderator introduced NGT and provided an overview of the steps involved in soliciting participants’ perspectives on barriers to CPG implementation. The moderator then stated: “Thinking about how clinical practice guideline recommendations are incorporated into routine practice, what are the most important barriers at your institution?” Participants generated ideas in silence. Then, each participant provided responses which were recorded on a flipchart.

Ideas raised during the first round were discussed to ensure that all participants understood each idea. Participants combined ideas they felt were similar or separated ideas containing more than one concept. This process was followed by a vote; each participant placed zero, one or more stickers beside each idea listed. The number of stickers (maximum 15) placed indicated their sense of each idea’s importance. After the first vote, participants explained and discussed their decisions. A second vote, identical in format to the first, was then conducted. Thus, a final ranking of items by importance according to the sum of second-round votes from all participants was created. These steps were repeated to identify CPG implementation facilitators.

In part 2, opinions about implementation of specific supportive care CPG recommendations were solicited. Depending on the time available, one or two strong recommendations from three COG-endorsed CPGs were discussed: CINV, FN, and FP. These CPGs were specifically chosen because, among the COG-endorsed supportive care CPGs available at the time, they describe common supportive care issues and participants were expected to have had experience providing care to patients with these conditions or needs. Participants generated ideas for implementation in silence and provided responses which were recorded on a flipchart.

Focus group sessions were audiotaped but not transcribed. A co-investigator took notes of the ideas discussed, the wording used and the tone of the group using a structured notes template similar to the moderator’s guide; another co-investigator documented the results of the NGT discussions.

### Analysis

Fisher’s exact tests were used to evaluate differences in characteristics of institutions that did and did not contribute focus group members.

Notes from each focus group were analyzed using a directed content analysis. Analysis was interpretive and descriptive. All domains of the implementability framework [8] of Gagliardi et al. were used to establish themes. This framework has been used to structure projects in CPG adaptation and implementation in various domains and settings including spinal cord injury rehabilitation in adults [23], exploration of perspectives on CPG uptake regarding medical procedures among adult and pediatric medical staff [24], and development of a medical laboratory response checklist [25]. It was chosen since it was developed specifically to facilitate CPG implementation rather than facilitate practice change in general. Operational definitions of framework themes were adapted by two co-investigators (AJS and LLD) as necessary to clarify meaning. They independently coded the CPG implementation facilitators and barriers identified in the first focus group using this framework. A third co-investigator (TL) entered and compared the coding using NVivo 12 (QSR International; Version 12.5.0.815 (64-bit), Edition: Plus). These co-investigators refined the operational definitions of the themes as necessary. For example, applicability was defined as pertaining to a patient or institution, not to an individual healthcare professional; “buy-in” of healthcare professionals was categorized as accommodation—user needs/values and the use of the category implementation—tools were applied to positive contexts only. Two co-investigators (AJS and LLD) then independently coded the notes generated by the remaining focus groups. New categories and sub-categories were coined to code concepts that did not fall within the pre-determined themes. Each item may have been coded to more than one theme.

The technique of McMillan et al. [26] was used to analyze the NGT findings across all focus groups. This approach considers the voting frequency (popularity of the item among all participants) and the relative importance (sum of the votes within each focus group) of the NGT items. NGT items were coded as described above. The total score of each theme was the sum of the second votes from NGT items coded under that theme. The top five scoring themes for each focus group were then assigned a ranking, with the highest scoring theme ranked as #1 and the lowest scoring theme ranked as #5. The total scores for each of the top five scoring themes from each focus group were then added together and ranked by relative proportion of votes to generate the top five scoring themes across all focus groups. A post hoc decision was made to present the NGT items within each theme according to the implementation strategy categories described by the NCI [27]. This was done by one co-investigator (LLD) and checked by another (AJS). Discrepancies were resolved by consensus.

The implementation steps identified by each focus group during part 2 were recorded. Facilitators and barriers to each of these steps identified by participants were categorized by two co-investigators (AJS and LLD) using the Implementability Framework [8] and then collated. These were used to illustrate the findings of part 1. Since some focus groups discussed only one CPG recommendation in part 2, data from the discussion of the first CPG recommendation presented to each focus group were analyzed.

## Results

Volunteer offers were received from healthcare professionals from 23 of the 26 institutions that opened ACCL15N1CD. Focus groups were held from March 2017 to October 2018. Saturation of ideas was reached after seven focus groups involving 35 participants from 18 institutions. The majority of participating institutions (61%) that contributed participants were stand-alone pediatric hospitals. Participating institutions were geographically dispersed across all five regions of the USA. However, the northeast and western regions were less well represented. Table 1 presents participant and focus group characteristics. Of the characteristics tested, only geographical site location differed between institutions that did and did not contribute focus group members (Additional file 2).

**Table 1:** Characteristics of the focus group participants and focus groups

Characteristic	
**Participant characteristics (*n*=35)**	
Female sex, *n* (%)	28 (80)
Profession, *n* (%)	
Physician	12 (34)
Nurse	10 (29)
Pharmacist	3 (9)
Psychologist	3 (9)
Other^a^	7 (20)
Median years since completion of most recent training (IQR)	15 (9–22)
Median years of pediatric oncology experience (IQR)	11 (5–17)
Median years at current institution (IQR)	8 (3–14)
Median percentage time spent providing direct patient care (IQR)	75 (75–90)
Self-assessed awareness of CPG, n (%)	
Very aware	14 (40)
Fairly aware	21 (60)
Not very aware	0
Not at all aware	0
**Focus group characteristics (*n*=7)**	
Focus group type, *n* (%)	
Mixed	5 (71)
Physician-only	1 (14)
Non-physician-only	1 (14)

*n* number, *IQR* interquartile range

^a^Other: clinical research coordinators/associates, dieticians, occupational therapists, physical therapists, and social workers

### Part 1

The prioritized themes and examples of corresponding NGT items are presented in Tables 2 and 3. Additional file 3 presents the total scores of the themes across all focus groups. The top five facilitators and barriers to CPG implementation across all focus groups are discussed below.

**Table 2:** CPG implementation facilitators: top five prioritized themes categorized by implementation strategy^18^ with NGT item examples

Facilitator theme	Examples of NGT items
1. **Organizational factors**	
Develop stakeholder interrelationships	Networking with others outside own institution
	Collaboration between and across disciplines
	Commitment to strive for best practice
Convene teams	Dedicated team members to pediatric oncology
	Strong physician CPG champion
	Designated person as implementation planner
	Inclusion of all involved in guideline-related care
Utilize financial strategies	Adequate resources and time available
Practice facilitation	CPGs are considered trustworthy as they are COG-endorsed
Support practitioners	Commitment/advocacy from physician leadership
Change infrastructure	Regular meetings to discuss CPGs
Train and educate stakeholders	Formal presentation at staff meetings
2. **Individual factors**	
Develop stakeholder interrelationships	Eagerness/willingness for consensus toward implementation/use
Convene teams	Goal of best patient care
Engage consumers	Family buy-in
Provide interactive assistance	Mentoring and education available among team members
Use evaluative and interactive strategies	Good patient outcomes encourage CPG
Support practitioners	Hospital/institution (upper management) supportive of change
Change infrastructure	Consistent practitioner
Train and educate stakeholders	Knowing the evidence to support the CPG
	Mentoring among team members
**3. User needs/values**	
Develop stakeholder interrelationships	Commitment/advocacy from physician leadership
Convene teams	Buy-in/engagement of all staff
Provide interactive assistance	Mentorship within discipline
Change infrastructure	Designated interdisciplinary committee to discuss and adopt guideline
**4. System factors**	
Develop stakeholder interrelationships	Patient-centered care—safety-focused
Convene teams	Supportive staff
Utilize financial strategies	Network that provides financial and administrative support (for example NCORP)
Engage consumers	Education to families/materials
Provide interactive assistance	EMR integration: standardized order sets, hard stops, prompting
Use evaluative and interactive strategies	Organized systematic approach to implementation
Support practitioners	Interdisciplinary functional team—good and open communication
Change infrastructure	Staff huddles
Train and educate stakeholders	Multidisciplinary rounds
**5. Implementation: strategies**	
Develop stakeholder interrelationships	Science-based approach
Practice facilitation	Peer pressure
Provide interactive assistance	Embedded in COG protocols with links
Use evaluative and interactive strategies	Metrics/goals
Support practitioners	Visible signs/reminders
	E-mail alerts regarding new CPG or updates
Change infrastructure	Negative reinforcement/monitoring board
Train and educate stakeholders	External continuing education
	Journal club to share practice change
	Regular tumor boards

**Table 3:** CPG implementation barriers: top five prioritized themes categorized by implementation strategy^18^ with NGT item examples

Barrier theme	Examples of NGT items
**1. Organizational factors**	
Develop stakeholder interrelationships	Conflict with institutional policies
	External and internal resource allocation: staff, space/geography, money and medication
	Coordinating between peds/adult services for AYAs
	Variability of institutions caring for same patient (inconsistency, HR, location, providers)
Convene teams	Lack of collaboration between disciplines within division
	Availability of personnel/resources/expertise related to CPG
Utilize financial strategies	Cost to implement change
	Access to specialists/meds to support CPG
Engage consumers	Lack of incentives to use CPGs
	Differences in “school of thought” and staff non-compliance with recommendations
Support practitioners	Time required electronic health record adaptation
Change infrastructure	Nursing care delivery: Staffing, ratio, and location
	Lack of consistency
	Continuity of process (external and internal)
Train and educate stakeholders	Lack of mandatory education for providers/staff in different departments
	New team members, staff turnover
	Education of outside providers
**2. Individual factors**	
Develop stakeholder interrelationships	Difference provider/team opinion and multiple providers in decision making
	Different practice styles/stubbornness/bad case (experience)
	Belief by some providers that they already know
	Health care professionals’ perceptions, experiences and standards of care
	Ability to “network” with other institutions around best practices
	Trust in CPGs
Convene teams	Lack of owner of CPG
Utilize financial strategies	Insurance coverage/limitations/prior authorizations
	Formulary restrictions
Engage consumers	Language/cultural differences and preferences
Adapt and tailor to context	Limitations of CPG for individual patient and lack of understanding regarding how to use CPG
Train and educate stakeholders	Lag in education of professionals regarding CPGs or updates
**3. System factors**	
Develop stakeholder interrelationships	Administration hurdles/hospital bureaucracy
	Non-oncology staff providing care
	Changing institutional culture is difficult
Convene teams	Time
Provide interactive assistance	Technical/electronic health record challenges causing delays
Support practitioners	High stress related to EHR
	Language/cultural differences and preferences
Change infrastructure	Speed of dissemination
	Lack of formal committee/procedure for CPG implementation
Adapt and tailor to context	No room for gray areas, conflicting guidelines
Train and educate stakeholders	Time required for training
**4. User needs/values**	
Develop stakeholder interrelationships	Concern following some aspects of CPG
	Conflict with a local approach/study
	Knowledge gaps between departments in same hospital
Convene teams	Not including other disciplines/team members in decision to follow/implement
Engage consumers	Family/patient resistance and family stress
**5. Professional** **(education, training, or competencies needed by clinician/staff to deliver recommendations)**	
Provide interactive assistance	Time required for electronic health record adaptation
Train and educate stakeholders	Keeping up with new literature
	Non-oncology staff providing care and educating other departments

#### CPG implementation facilitators

1. **Organizational factors**: Participants spoke of the importance of “institutional buy-in” to the concept of CPG-consistent care delivery, describing it as an “eagerness” to build consensus for CPG implementation among multiple departments, divisions, and professionals. This was further demonstrated by the resources allocated to CPG implementation. Key among these resources was the CPG champion, “a person who is interested, willing, and eager,” who would, as the “owner” of the CPG implementation process, develop consensus, rally resources, develop processes, and measure success. In addition, practical, logistical support (e.g., dedicated time for implementation activities) provided by the organization helps “keep people in tune with the guidelines.”
2. **Individual factors**: A supportive attitude of all those involved in care delivery (healthcare professionals, other staff, family members, patients) was believed to be critical. Participants identified an individual’s philosophy of evidence-based care, their knowledge of the CPG being implemented, and their previous positive experiences with CPG-consistent care as facilitators.
3. **User needs or values:** The extent to which healthcare professionals value care standardization was raised as a specific attribute that would facilitate CPG implementation.
4. **System factors**: An organized, systematic approach to CPG implementation that included education of stakeholders within and external to pediatric oncology was favored. Participants suggested that specific CPG implementation tools be created (e.g., standardized order sets, prompts toward CPG-consistent orders, electronic health record (eHR) integration). Goal setting and metrics for implementation outcomes were also felt to be important.
5. **Implementation strategies**: Three focus groups offered suggestions for CPG implementation methods distinct from other facilitators, such as COG endorsement, communication strategies, and demonstration of benefits of CPG-consistent care.

#### CPG implementation barriers

1. **Organizational factors**: Examples of lack of institutional support of CPG-consistent care raised by participants were inadequate resourcing of CPG implementation and bureaucratic hurdles that lead to lengthy CPG implementation periods. Further, participants spoke of barriers due to the costs of the new care delivery paradigm that CPG implementation may represent such as access to specialists, non-formulary medications, or specialized interventions. Lastly, an organizational culture that tolerates siloed communication and hinders collaboration among and between stakeholders was identified as a barrier.
2. **Individual factors**: Participants discussed the effects of a negative attitude toward change or CPG-consistent care: the beliefs of some healthcare professionals that “they already know” and that “change is not necessary.” Since trust in CPGs is variable, building consensus around CPG-consistent care is challenging. Lack of or lagging education of patients, families, and healthcare providers with respect to the CPG, the evidence informing it or how to use it were also identified as barriers. Participants felt that this was particularly troublesome when CPG implementation involved personnel external to pediatric oncology. Participants also stated that the language, cultural preferences, and values of stakeholders may present barriers if they are not addressed proactively during CPG implementation.
  A patient’s inability to access resources (e.g., insurance coverage limitations, transportation costs, formulary restrictions) was also raised as a barrier to CPG-consistent care. One participant spoke of the futility felt by healthcare professionals when they offer an expensive CPG-consistent intervention to families without being able to offer financial support: “It’s like telling someone about Mars. You can never go there. But it would be really good for you if you could.”
3. **System factors**: Participants often spoke of the ineffectiveness of their institution’s eHR or order entry system to support CPG-consistent care. One participant stated: “eHR template build-outs slow us down.” The lack of a specified pathway for CPG implementation and difficulties in accessing CPGs were also raised as barriers.
4. **User needs and values**: Some participants felt that physicians did not want or appreciate non-physician contribution to CPG implementation. They also felt that some non-physician healthcare professionals may consider CPG implementation to be outside their role. In either case, CPG implementation was believed to suffer. In pediatric institutions that exist within mixed institutions (i.e., adult and pediatric), institutional values and priorities may be driven by the needs of adult patients. Consequently, pediatric CPG implementation can be obstructed. Difficulty adding drugs to the formulary that are used solely by children and adolescents was raised as an example.
5. **Professional:** Participants mentioned that maintaining competencies (e.g., keeping up with the published literature on each CPG topic) needed to deliver CPG-consistent care was an impediment to CPG implementation. Similarly, delays in providing CPG-focused education during CPG implementation were also thought to be limiting.

### Part 2

Implementation of CPG recommendations on FP, FN, and CINV were discussed by four, two, and one focus groups, respectively. Five to 12 implementation steps per CPG were ranked by each focus group (Additional file 4). Aspects of CPG implementation highlighted in these discussions are presented below.

The organization’s role in networking with other organizations was especially important when resources required for CPG implementation were shared or contracted out (e.g., FP). However, participants felt that organizations deprioritized CPG-consistent supportive care when CPG implementation outcomes were not sentinel or reportable events (e.g., CINV control).

A healthcare professional’s culture and beliefs and their exposure to other cultures were felt to influence supportive care CPG implementation. Similarly, patient and family values were highlighted as significant potential barriers. This was most often raised during discussion of FP CPG implementation.

Participants stated that integration within existing systems could enhance CPG implementation. Examples were embedding drugs (e.g., CINV, FN) and specialist referrals in order sets (e.g., FP). When used in this way, order sets can encourage CPG-consistent decision making by clearly outlining CPG-consistent care that is adapted to the local context. Conversely, systems can prevent timely CPG implementation: “if they can’t make the change for a year then you kind of have to come up with work-arounds.”

The need to address funding issues when planning CPG implementation was keenly felt when the costs of CPG-consistent care fall directly on families. For example, without funding in place, participants were reluctant to offer CPG-consistent FP care.

## Discussion

Using the focus group and NGT methodologies, we found that healthcare professionals in pediatric NCORP institutions believe organizational factors to be critical to supportive care CPG implementation. They look to their organization to cultivate strong relationships between internal and external stakeholders and allocate resources to initiate and sustain the changes required by CPG-consistent care. Conversely, they believe that CPG implementation is more difficult when organizations do not value evidence-based practice, care standardization, or collaboration.

Consistent leadership, existence of multi-disciplinary teams, positive perceptions of CPG usefulness, and provision of CPG-specific training early in implementation were identified as common facilitators of CPG implementation by a recent metareview of CPG barriers and facilitators [12]. Common barriers identified were the absence of a leader, lack of time, lack of CPG clarity, and lack of awareness that the CPG existed. Bierbaum et al. undertook a systematic review of adult oncology clinicians’ perceptions regarding facilitators and barriers to CPG adherence [28]. Clinicians’ concerns regarding CPG integrity and negative perceptions of CPG-consistent care were significant implementation barriers. Identified facilitators included a belief in the relevance of the CPG.

Similar facilitators and barriers to CPG implementation have been identified in pediatric settings. In a systematic review of CPG implementation in pediatric palliative care, common barriers were training of healthcare professionals and formation of multi-disciplinary teams [29]. Pediatric rehabilitation therapists felt that clinician confidence, resource availability, and organizational support were important to CPG implementation [30].

Due to the emphasis on protocolized treatment in pediatric oncology, we had speculated that our participants might approach CPGs differently than other healthcare professionals. However, with respect to barriers and facilitators of CPG implementation, our participants’ perspectives are similar to those of healthcare professionals in other specialties. Our participants emphasized the importance of the organization in CPG implementation, citing its role in priority setting, establishing a CPG implementation structure and resource provision. Participants spoke of an ideal organizational culture where CPG implementation was one of many ways of nurturing evidence-based, interprofessional, interdisciplinary, and patient-focused care. They also spoke of the importance of a formal champion appointed and resourced by the organization with accountability for CPG implementation from its inception. This role is distinct from the use of a champion as a dissemination strategy [31, 32].

Within the implementability framework of Gagliardi et al., [8] themes relating to the CPG itself (e.g., usability, adaptability, validity, applicability, and communicability) were not prioritized by our participants. COG endorsement may serve to allay concerns relating to CPG usability and validity. Similarly, the pre-determined focus on supportive care CPG implementation perhaps deprioritized the need to evaluate the CPG purpose (accommodation). Interestingly, participants believed the role of systems (accommodation: technical) such as the eHR to be less important to successful supportive care CPG implementation.

Supportive care CPGs are relatively new to pediatric oncology [33]. It is possible that the barriers described by participants reflect, at least in part, incomplete acceptance of the importance of supportive care itself by pediatric oncology leadership. It is likely that efforts to implement supportive care CPGs in pediatric oncology must overcome barriers pertinent to both supportive care as well as to CPGs. We speculate that capturing the attention of organizational leadership so that CPG-consistent supportive care is prioritized may be particularly difficult in pediatric oncology.

Our participants outlined implementation plans for specific CPGs (Additional file 4). This information can be viewed as a preliminary implementation framework that is meaningful to pediatric NCORP sites. Although implementation details may vary considerably depending on the CPG topic, cross-cutting suggestions include prioritization of CPG-consistent care by the institution and provision of resources including staff time, development of an interprofessional implementation team led by a champion, patient and family involvement, development of implementation tools, education of all stakeholders, and feedback on adherence and impact of CPG-consistent care.

Strengths of our study include qualitative methodology and participation of a variety of healthcare professionals from several types of geographically dispersed NCORP institutions. Limitations may include participant bias in favor of CPG implementation. This concern is diminished by the broad range of implementation facilitators and barriers identified. Our methods did not allow evaluation of potential differences in perspectives between different types of health care professionals. In addition, we do not know if non-participating institutions differed from participating institutions with respect to CPG implementation experience. Further, the ability to generalize our findings to non-NCORP institutions is uncertain. However, participation of a high proportion of health care professionals from academic NCORP sites may increase the applicability of our findings to non-NCORP institutions since they tend to be academic institutions. Lastly, our study did not evaluate the influence of institutional characteristics (e.g., size, type, location, and culture) or institution-level resources on perceptions regarding facilitators and barriers to CPG implementation.

## Conclusions

Contemporary pediatric cancer treatments are intense, and treatment-related symptoms are common [34–36]. In part, symptom control is related to delivery of CPG-consistent supportive care [37]. Healthcare professionals at pediatric NCORP institutions believe that organizational factors are the most important determinants of supportive care CPG implementation. Specifically, they believe that CPG-consistent supportive care is most likely to be delivered in organizations that prioritize evidence-based care, provide structure and resources to implement CPGs, and eliminate CPG implementation barriers. In this, they are similar to healthcare professionals in other settings. However, given the prioritization of cancer treatment protocols over supportive care CPGs in pediatric oncology, we expect that successful implementation of CPG-consistent supportive care requires tactics aimed at influencing organizational priorities and resource allocation. Our work suggests a practical approach to the implementation of supportive care CPGs in pediatric oncology. Future work should explore the impact of interventions that leverage facilitators of and overcome barriers to CPG-consistent supportive care implementation and describe resultant outcomes in pediatric oncology patients.

## Supplementary Information

**Additional file 1.** Focus group moderator’s guide

**Additional file 2.** Characteristics of institutions that did and did not contribute focus group participants

**Additional file 3.** Facilitators and barriers to clinical practice guideline implementation: Top five prioritized themes within each focus group and across all focus groups.

**Additional file 4.** Clinical practice guideline implementation steps identified by each focus group in Part 2.

## Data Availability

No datasets are available from this study owing to the consents given by participants, which limits data to the research team only.

## Abbreviations

COG: Children’s Oncology Group
CPG: Clinical Practice Guideline
NCI: National Cancer Institute
NCORP: NCI Community Oncology Research Program
NGT: Nominal Group Technique
