# Supplementary material for: Facilitators and barriers to clinical practice guideline-consistent supportive care at pediatric oncology institutions: a Children’s Oncology Group study
Source: Implement Sci Commun. 2021 Sep 16;2:106. doi: 10.1186/s43058-021-00200-2 (PMC8447588; doi:10.1186/s43058-021-00200-2)
Supplement: Supplementary file 1 — Additional file 1. Focus group moderator’s guide [file 43058_2021_200_MOESM1_ESM.pdf]

Sugalski AJ et al. Facilitators and barriers to clinical practice guideline-consistent supportive care at pediatric oncology institutions: A Children's Oncology Group study. Implement Sci Commun 2021

Additional file 1: Focus group moderator guide

ACCL15N1CD FOCUS GROUP MODERATOR GUIDE version 3

DATE: ____________________________________ Location: ____________________________________

Moderator Pt 1: ______________________________ Moderator Pt 2: ______________________________

Recorder 1 (content): __________________________ Recorder 2 (mood): __________________________

Accountant: ________________________________ Logistics Coordinator: ________________________

Flip chart writer Pt 1: ________________________ Flip chart writer Pt 2: ________________________

Flip chart ass't Pt 1: _________________________ Flip chart ass't Pt 1: _________________________

Focus Group Type: Inter-professional ☐ Physician-only ☐ non-Physician-only ☐

**Ideal Running Time:**	**5 hr**	**Actual Running Time:**	**0 min**
**Ideal Start Time:**	**o'clock**	**Actual Start Time:**	**o'clock**

PART 1

Good morning/afternoon, I am [name] - a member of the Children's Oncology Group and the moderator for the first part of this session.

Today you will be participating in a focus group for COG study ACCL15N1CD, Use of Evidence-Based Supportive Care Clinical Practice Guidelines in Pediatric Oncology. This focus group should take 4 to 5 hours including a meal break. We are holding focus groups like this one to learn more about facilitators and barriers to the implementation of guideline-consistent care.

This focus group discussion will be audio-recorded.

Your participation is completely voluntary. If you do not wish to participate, you may stop or leave the group at any time without penalty. You may also skip any questions that you do not want to answer. Your responses in this focus group will be used anonymously in publication. Taking part in this focus group is your agreement to participate.

This research has been reviewed and approved by the NCI Pediatric Central Institutional Review Board.

If you would like a copy of this script for your records, please let me know and I will provide one to you. If you have any questions regarding this research, contact the ACCL15N1CD Study Chairs, Drs. Lee Dupuis and Aaron Sugalski. If you have any questions regarding your rights as a research subject, please contact the NCI Central IRB.

In addition to me, [names] will be present to observe and take notes on the discussion and [names] are here to provide logistical support and . . . .

Do you agree for the data collected in the study to be used anonymously in publication?

Do you also agree to have the focus group discussion audio recorded?

Do you have any questions before we proceed?

Our guidelines for this afternoon are: [show slide], review and flip chart writer will post on wall.

1. Let's take a minute to introduce ourselves [study committee and focus group members] before we get started.
   Let's go around the table and please say:

   a) your name,

   b) where you live,

   c) the name of your institution and how long you've worked there

   d) what you did for fun this past weekend (as ice-breaker)

Thank you! Today we will be discussing clinical practice guideline implementation. [show slides] Clinical practice guidelines are . . . For several years the COG has reviewed available supportive care clinical practice guidelines and endorsed those which deal with topics relevant to the COG and meet our standards for rigor of development. The COG-endorsed supportive care guidelines are available on the website . . .

We will be using nominal group technique to discuss factors that influence **supportive care** clinical practice guideline implementation at NCORP sites. This technique helps make sure that everyone's opinions are heard. To be fully transparent, here are some ground rules for our discussion. [slide]

I will ask the entire group the first of 2 questions and then ask each of you to think of your responses in silence. If you want, you may write your own responses to the question on the paper provided. Then everyone's responses will be written on the flip chart. We will discuss these responses together to make sure that they are clear.

You will see that you each have 2 groups of 15 stickers. You will use one group of 15 stickers to vote on which of these responses are the most important to you. You can put more than one sticker on a single response – in fact you can put all 15 stickers on 1 response if you want! We will then count the number of stickers people have placed beside each item and then freely discuss the rankings.

Everyone will then vote again on which responses are most important once more using the second group of 15 stickers and a rank ordered list of responses will be created using the sum of each response's votes.

We will then repeat the process with a second and last question.

After that we will break to eat and reconvene to discuss implementation of specific guideline recommendations.

You may leave the focus group at any time to take a break or to get a drink from the table. **[Name of logistics person] – would you please tell us where the women's and men's restrooms are?**

Do you have any questions about the process?

Let's talk a bit about supportive care clinical practice guidelines then!

1. **Please tell me about how supportive care CPGs are normally incorporated into care at your institution.**

   (Suggested Probes: Is it different for physicians, nurses, etc? Is it different depending on the CPG topic?)

2. **How often do you actually use a CPG in your practice to direct patient care?**

   (Suggested Probes: when caring for patients? discussing care with colleagues? teaching?)

3. **How do you feel about the way that CPGs fit within your daily workflow?**

4. **How you feel about having the CPGs available in an electronic format?**

   - **Some examples are the COG website, the e-health record or as a stand-alone website or 'app'**

**Ideal Running Time: 30 min**	**Actual Running Time: min**
**Ideal Time: o'clock**	**Actual Time: o'clock**

Question #1: [show slide]

Please think about your answer to the following question silently:

*Thinking about how clinical practice guideline recommendations are incorporated into routine practice, what are the most important **barriers** at your institution?*

5 MINUTES OF SILENCE

**Ideal Running Time: 35 min**<br>**Ideal Time: o'clock**	**Actual Running Time: min**<br>**Actual Time: o'clock**

Please share your responses! Let's go around the table. If all your points have been raised or if your point is similar to one that has already been raised, feel free to 'pass'.

Moderator guides conversation so that each focus group member offers barriers until all thoughts are exhausted. The recorder writes each thought on the flip chart.

Let's review the list of items that we have generated. The number of items doesn't matter – we can have as many or as few as you want. But . . .

- **Are any so similar that they can be combined without losing any meaning?**
- **Should any one of the items be split into more than 1 idea?**
- **Is there anything missing?**

**Ideal Running Time: 55 min**<br>**Ideal Time: o'clock**	**Actual Running Time: min**<br>**Actual Time: o'clock**

Let's each silently re-read what you've identified as barriers to clinical practice guideline implementation.

Is there anything else anyone would like to share about barriers to guideline implementation at their institution?

VOTE #1.1

Please take 15 stickers (type #1) and walk up to place stickers beside the responses which are most important to you. Feel free to put any number of stickers (1 to 15) beside 1 response.

**Ideal Running Time: 1 hr 10 min**<br>**Ideal Time: o'clock**	**Actual Running Time: min**<br>**Actual Time: o'clock**

Accountant counts stickers/item and adds count to the flip chart page.

Let's review your votes. Moderator reads out items in rank order.

So, it seems like you are saying that [item], [item] and [item] are important to this group.

Let's discuss some reasons for your selections. Which items are most important to you and why?

Remember that it is fine to disagree and that there is no right answer.

Moderator guides discussion re reasons for rankings.

Does this ranking reflect your personal experience?

VOTE #1.2

Please take 15 stickers (type #2) and walk up to place stickers beside the responses which are most important to you. Feel free to put any number of stickers (1 to 15) beside 1 response.

**Ideal Running Time:**	**1 hr 25 min**	**Actual Running Time:**	**min**
**Ideal Time:**	**o'clock**	**Actual Time:**	**o'clock**

Time to stand and stretch?

Question #2: [show slide]

Time to move on to our second question:

Please think about your answer to this question silently:

Thinking about how clinical practice guideline recommendations are incorporated into routine practice, what are the most important facilitators at your institution?

5 MINUTES OF SILENCE

**Ideal Running Time:**	**1 hr 35 min**	**Actual Running Time:**	**min**
**Ideal Time:**	**o'clock**	**Actual Time:**	**o'clock**

Please share your responses! Let's go around the table. Remember, if all your points have been raised, feel free to 'pass'.

Moderator guides conversation so that each focus group member offers facilitators until all thoughts are exhausted. The recorder writes each thought on the flip chart.

Let's review the list of items that we have generated. Again, the number of items doesn't matter – we can have as many or as few as you want. But . . .

- **Are any so similar that they can be combined without losing any meaning?**

- **Should any one of the items be split into more than 1 idea?**

- **Is there anything missing?**

**Ideal Running Time: 1 hr 50 min**<br>**Ideal Time: o'clock**	**Actual Running Time: min**<br>**Actual Time: o'clock**

Let's each silently re-read what you've identified as barriers to clinical practice guideline implementation.

Is there anything else anyone would like to share about facilitators of clinical practice guideline implementation at your institution?

VOTE #2.1

Please take 15 stickers (type #1) and walk up to place stickers beside the responses which are most important to you. Feel free to put any number of stickers (1 to 15) beside 1 response.

**Ideal Running Time: 2 hrs**<br>**Ideal Time: o'clock**	**Actual Running Time: min**<br>**Actual Time: o'clock**

Accountant counts stickers/item and adds count to the flip chart page.

Let's review your votes. Moderator reads out items in rank order.

So, it seems like you are saying that [item], [item] and [item] are important to this group.

Let's discuss some reasons for your selections. Which one is most important to you and why? Remember that it is fine to disagree and that there is no right answer.

Does this ranking reflect your personal experience?

VOTE #2.2

Please take 15 stickers (type #2) and walk up to place stickers beside the responses which are most important to you. Feel free to put any number of stickers (1 to 15) beside 1 response.

**Ideal Running Time: 2 hrs 15 min**<br>**Ideal Time: o'clock**	**Actual Running Time: min**<br>**Actual Time: o'clock**

MEAL BREAK – We will break for an hour. Please help yourself to lunch/supper and we will reconvene at [state time].

Moderator and recorders will move to a private room to review NGT results so that the results can be brought into the discussion of specific guideline recommendations. Group will also choose order of CPG recommendations to be discussed in part 2.

PART 2

**Ideal Running Time:**	**3 hrs 15 min**	**Actual Running Time:**	**min**
**Ideal Time:**	**o'clock**	**Actual Time:**	**o'clock**

Thank you for returning for the second half of our session! My name is [name] and I will be your moderator for the second part of today's session.

We are going to get specific now and zero in on a few recommendations of current COG endorsed supportive care clinical practice guidelines.

Recommendation #1

[Show a CPG recommendation on screen.]

Here is the recommendation that we will discuss. It was developed by [the international febrile neutropenia guideline panel/Pediatric Oncology Group of Ontario/ASCO] and is a strong recommendation. This means that the guideline development panel felt that there was little uncertainty about outcomes when care is delivered according to the recommendation. In fact, strong recommendations may be considered as policies or standards.

Please think about your answer to this question silently:

Imagine that you want to implement this recommendation at your institution. What steps would you take to do this?

Feel free to write down your thoughts on paper.

5 MINUTES OF SILENCE

Let's go around the table and share the steps of your plans.

If all your points have been raised or if your point is similar to one that has already been raised, feel free to 'pass'.

What would be your first step to implement this guideline recommendation?

Moderator guides conversation so that each focus group member offers implementation steps until all thoughts are exhausted. Implementation steps are noted on the flip chart – **paper should be pre-formatted to allow space for barriers and facilitators for each step.**

For each implementation step, moderator probes regarding possible facilitators and barriers and solutions to the possible barriers identified.

The recorder writes each thought/item on the flip chart and captures implementation step, facilitators, barriers, and solutions.

Probes:

- raise issues discussed in part 1: Would [issue] play a role in the implementation plan?

- what do people currently do re [recommendation]? How do they feel about it?

- do you think that people generally agree with the recommendation? If not, how do you feel that consensus could be built?

- do you feel that people already have the skills they need to implement this recommendation? If not how could they get them? e.g. continuing education, training modules.

- do you feel that your care teams are organized in a way that might influence the implementation of this recommendation?

- how could your information system (e.g. e-HR, CPOE) impact the implementation of this recommendation?

- do you feel that having the recommendation available electronically (e.g. COG website) might help with implementation?

- once implemented what do you feel could be put into place to encourage people to continue to provide care according to the recommendation?

It seems that this group is saying that [item], [item], [item] would be important steps to the implementation of this recommendation.

Does anyone have anything to add or clarify?

**Ideal Running Time:**	**3 hr 45min**	**Actual Running Time:**	**min**
**Ideal Time:**	**o'clock**	**Actual Time:**	**o'clock**

VOTE Recommendation #1

You will see that you have 3 sets of stickers in front of you. Each set of stickers is numbered 1 through 15. Please take 1 set of 15 stickers. Walk up to place a sticker beside the implementation steps you would use to implement this guideline. Place sticker #1 beside the first step you would take, sticker #2 beside the 2nd step, etc. You don't have to put a sticker beside each item on the chart and you don't have to use all your stickers. Just use the stickers to number the steps that you would use to implement this guideline.

Let's review your votes. Moderator reads out items in rank order.

So, it seems like you are saying that [item], [item] and [item] is the order of implementation that you think would be helpful.

Moderator guides discussion re barriers and facilitators of the steps using above-listed probes as necessary. Information is noted on the flip chart.

- **In your opinion, what are the important potential barriers to the success of this plan at your institution?**

- **How can these important barriers be overcome?**
  - **Probes:**
    - **How would you reduce the impact of [barrier]?**

- **In your opinion, what are the important potential facilitators of the success of this plan at your institution?**

  - **Probes:**

    - **How can these facilitators be leveraged?**
    - **What incentives could be provided to increase the chance of people that people will continue to provide care according to the guideline recommendation? e.g**. personal recognition or appreciation (from managers, colleagues or the community), continuing education (opportunities to participate in educational activities), working conditions (facilities, equipment or security), career development (possibilities to specialise or be promoted), management (working relationship with managers)

**Ideal Running Time: 4 hr 10 min**<br>**Ideal Time: o'clock**	**Actual Running Time: min**<br>**Actual Time: o'clock**

Recommendation #2

Thank you for that discussion! Now we're going to jump topics and talk about how you would plan the implementation of another guideline recommendation.

[Show a CPG recommendation on screen.]

Here is the next recommendation that we will discuss. It was developed by [the international febrile neutropenia guideline panel/POGO/ASCO] and is a strong recommendation - meaning that the guideline development panel felt that there was little uncertainty about outcomes when care is delivered according to the recommendation. In fact, strong recommendations may be considered as policies or standards.

Please think about your answer to this question silently:

Imagine that you want to implement this recommendation at your institution. For CPG recommendation #1 you had said that the implementation steps ordered on the flip chart here would be important.

What would the steps in your implementation plan look like?

How are the implementation steps for this second CPG recommendation different? How are they the same?

Feel free to write down your thoughts on paper.

5 MINUTES OF SILENCE

Let's go around the table and share your plans.

If all your points have been raised if your point is similar to one that has already been raised, feel free to 'pass'.

What would be your first step to implement this guideline recommendation?

Moderator guides conversation so that each focus group member offers implementation steps until all thoughts are exhausted.

For each implementation step, moderator probes regarding possible facilitators and barriers and solutions to the possible barriers identified.

The recorder writes each thought/item on the flip chart and captures steps, barriers, facilitators and solutions.

Probes:

- raise issues discussed in part 1: Would [issue] play a role in the implementation plan?

- what do people currently do re [recommendation]? How do they feel about it?

- do you think that people generally agree with the recommendation? If not, how do you feel that consensus could be built?

- do you feel that people already have the skills they need to implement this recommendation? If not how could they get them? e.g. continuing education, training modules.

- do you feel that your care teams are organized in a way that might influence the implementation of this recommendation?

- how could your information system (e.g. e-HR, CPOE) impact the implementation of this recommendation?

- do you feel that having the recommendation available electronically (e.g. COG website) might help with implementation?

- once implemented what do you feel could be put into place to encourage people to continue to provide care according to the recommendation?

It seems that this group is saying that [item], [item], [item] would be important steps to the implementation of this recommendation.

Does anyone have anything to add or clarify?

**Ideal Running Time:**	**4 hrs 35 min**	**Actual Running Time:**	**min**
**Ideal Time:**	**o'clock**	**Actual Time:**	**o'clock**

VOTE Recommendation #2

Please take a set of numbered stickers. Walk up to place a sticker beside the implementation steps you would use to implement this guideline. Place sticker #1 beside the first step you would take, sticker #2 beside the 2nd step, etc. You don't have to put a sticker beside each item on the chart and you don't have to use all your stickers. Just use the stickers to number the steps that you would use to implement this guideline.

Let's review your votes. Moderator reads out items in rank order.

So, it seems like you are saying that [item], [item] and [item] is the order of implementation that you think would be helpful.

Moderator guides discussion re barriers and facilitators of the steps using above-listed probes as necessary.

- **In your opinion, what are the important potential barriers to the success of this plan at your institution?**

- **How can these important barriers be overcome?**
  - **Probes:**
    - **How would you reduce the impact of [barrier]?**

- **In your opinion, what are the important potential facilitators of the success of this plan at your institution?**
  - **Probes:**
    - **How can these facilitators be leveraged?**
    - **What incentives could be provided to increase the chance of people that people will continue to provide care according to the guideline recommendation? e.g**. personal recognition or appreciation (from managers, colleagues or the community), continuing education (opportunities to participate in educational activities), working conditions (facilities, equipment or security), career development (possibilities to specialise or be promoted), management (working relationship with managers)

**Ideal Running Time:**	**4 hrs 55 min**	**Actual Running Time:**	**min**
**Ideal Time:**	**o'clock**	**Actual Time:**	**o'clock**

Recommendation #3

To be considered only if time and energy of focus group participants allow.

Thank you for that discussion! Now we're going to jump topics again and talk about how you would plan the implementation of another guideline recommendation.

[Show a CPG recommendation on screen.]

Here is the recommendation that we will discuss. It was developed by [the international febrile neutropenia guideline panel/Pediatric Oncology Group of Ontario/ASCO] and is a strong recommendation. This means that the guideline development panel felt that there was little uncertainty about outcomes when care is

delivered according to the recommendation. In fact, strong recommendations may be considered as policies or standards.

Please think about your answer to this question silently:

Imagine that you want to implement this recommendation at your institution. For CPG recommendations #1 and #2 you had said that the implementation steps ordered on the flip chart here would be important.

What would the steps in your implementation plan look like?

How are the implementation steps for this third CPG recommendation different? How are they the same?

Feel free to write down your thoughts on paper.

5 MINUTES OF SILENCE

Let's go around the table and share your plans.

If all your points have been raised if your point is similar to one that has already been raised, feel free to 'pass'.

What would be your first step to implement this guideline recommendation?

Moderator guides conversation so that each focus group member offers implementation steps until all thoughts are exhausted. For each implementation step, moderator probes regarding possible facilitators and barriers and solutions to the possible barriers identified. The recorder writes each thought/item on the flip chart.

Probes:

- raise issues discussed in part 1: Would [issue] play a role in the implementation plan?

- what do people currently do re [recommendation]? How do they feel about it?

- do you think that people generally agree with the recommendation? If not, how do you feel that consensus could be built?

- do you feel that people already have the skills they need to implement this recommendation? If not how could they get them? e.g. continuing education, training modules.

- do you feel that your care teams are organized in a way that might influence the implementation of this recommendation?

- how could your information system (e.g. e-HR, CPOE) impact the implementation of this recommendation?

- do you feel that having the recommendation available electronically (e.g. COG website) might help with implementation?

- once implemented what do you feel could be put into place to encourage people to continue to provide care according to the recommendation?

It seems that this group is saying that [item], [item], [item] would be important steps to the implementation of this recommendation.

Does anyone have anything to add or clarify?

VOTE Recommendation #3

Please take 1 set of numbered stickers. Walk up to place a sticker beside the implementation steps you would use to implement this guideline. Place sticker #1 beside the first step you would take, sticker #2 beside the 2nd step, etc. You don't have to put a sticker beside each item on the chart and you don't have to use all your stickers. Just use the stickers to number the steps that you would use to implement this guideline.

**Ideal Running Time: hr min**<br>**Ideal Time: o'clock**	**Actual Running Time: min**<br>**Actual Time: o'clock**

So, it seems like you are saying that [item], [item] and [item] is the order of implementation that you think would be helpful.

Moderator guides discussion re barriers and facilitators of the steps using above-listed probes as necessary.

- **In your opinion, what are the important potential barriers to the success of this plan at your institution?**

- **How can these important barriers be overcome?**
  - **Probes:**
    - **How would you reduce the impact of [barrier]?**

- **In your opinion, what are the important potential facilitators of the success of this plan at your institution?**
  - **Probes:**
    - **How can these facilitators be leveraged?**
    - **What incentives could be provided to increase the chance of people that people will continue to provide care according to the guideline recommendation? e.g**. personal recognition or appreciation (from managers, colleagues or the community), continuing education (opportunities to participate in educational activities), working conditions (facilities, equipment or security), career development (possibilities to specialise or be promoted), management (working relationship with managers)

We’ve just talked a lot about factors that might help or hinder guideline implementation and adherence. We will summarize all your ideas and comments, bring them together with those that are generated by other focus groups and will use this information to develop trials to assess the impact of specific strategies to enhance guideline implementation.

This is the end of our session. **You have been a wonderful focus group!** We would welcome any comments you have about your experience today or suggestions for improvement. Thank you all very much for your work today and for your enthusiasm!

**Ideal Running Time: 5 hrs**<br>**Ideal Time: o’clock**	**Actual Running Time: min**<br>**Actual Time: o’clock**

Appendix

CPG Recommendations for Discussion in Part 2

1. Discontinue empiric antibiotics in patients who have negative blood cultures at 48 hours, who have been afebrile for at least 24 hours, and who have evidence of marrow recovery

2. Children receiving antineoplastic agents of moderate emetogenicity should receive: Ondansetron or granisetron plus dexamethasone

3. Sperm cryopreservation: Sperm cryopreservation is effective, and health care providers should discuss sperm banking with post-pubertal males receiving cancer treatment.
