# Supplementary material for: Facilitators and barriers to clinical practice guideline-consistent supportive care at pediatric oncology institutions: a Children’s Oncology Group study
Source: Implement Sci Commun. 2021 Sep 16;2:106. doi: 10.1186/s43058-021-00200-2 (PMC8447588; doi:10.1186/s43058-021-00200-2)
Supplement: Supplementary file 2 — Additional file 2. Characteristics of institutions that did and did not contribute focus group participants [file 43058_2021_200_MOESM2_ESM.pdf]

Additional file 2: Characteristics of institutions that did and did not contribute focus group participants

Characteristic	Participating Institutions (n=18)	Non-participating Institutions (n=8)	p value
Site Location, n (%)			
Western US	1 (6)	2(25)	
Southwestern US	5 (28)	0	
Northeastern US	1 (6)	0	0.046
Southeastern US	6 (33)	6 (75)	
Midwestern US	5 (28)	0	
Site Type, n (%)			
Minority/Underserved Community* (vs. Other)	9 (50)	2 (25)	0.39
Pediatric (vs. Mixed Adult and Pediatric)	11 (61)	6 (75)	0.67
Private (vs. Academic)	12 (67)	7 (88)	0.37

n: number

* Serve a population composed of at least 30% racial or ethnic minorities or rural patients.
