# Supplementary material for: Facilitators and barriers to clinical practice guideline-consistent supportive care at pediatric oncology institutions: a Children’s Oncology Group study
Source: Implement Sci Commun. 2021 Sep 16;2:106. doi: 10.1186/s43058-021-00200-2 (PMC8447588; doi:10.1186/s43058-021-00200-2)
Supplement: Supplementary file 3 — Additional file 3. Facilitators and barriers to clinical practice guideline implementation: Top five prioritized themes within each focus group and across all focus groups. [file 43058_2021_200_MOESM3_ESM.pdf]

Additional file 3: Facilitators and barriers to clinical practice guideline implementation: Top five prioritized themes within each focus group and across all focus groups. Only physicians participated in Focus Group (FG) 3 while only non-physicians participated in FG 2. FG 1 and 4 to 7 were mixed.

Theme	FG1	FG2	FG3	FG4	FG5	FG6	FG7	Total Score
**Facilitators**								
Implementation: Facilitators - System	40	10	17	14	33	17		131
Implementation: Facilitators - Organizational	33	38	16	41	54	84	42	308
Facilitators – Individual	31	26	26	11	40	84	40	258
Implementation: Strategies	15		32				19	66
Accommodation: User needs/values	14		6	38	11	51	22	142
Implementation: Tools		16				18	19	
Validity: Evidence Graded		10						
Accommodation: Human Resources					21			
Accommodation: Professional				14				
**Barriers**								
Implementation: Barriers - Organizational	36	25	30	25	46	76	32	270
Implementation: Barriers – Individual	24	30	43	26	13	45	38	219
Accommodation: Professional	17				13		16	46
Implementation: Barriers - System	14	24	22	16	26	34	18	154
Accommodation: User needs/values	10	8	13	18	31	29		109

Accommodation: Costs		9						
Applicability: Individualization			13					
Validity: Evidence Graded				21				
Accommodation: Human Resources					14			
Accommodation: Objective						22		
Implementation: Tools							19	
