# Supplementary material for: Facilitators and barriers to clinical practice guideline-consistent supportive care at pediatric oncology institutions: a Children’s Oncology Group study
Source: Implement Sci Commun. 2021 Sep 16;2:106. doi: 10.1186/s43058-021-00200-2 (PMC8447588; doi:10.1186/s43058-021-00200-2)
Supplement: Supplementary file 4 — Additional file 4. Clinical practice guideline implementation steps identified by each focus group in Part 2. [file 43058_2021_200_MOESM4_ESM.pdf]

Additional file 4: Clinical practice guideline implementation steps identified by each focus group in Part 2. Only physicians participated in Focus Group (FG) 3 while only non-physicians participated in FG 2. FG 1 and 4 to 7 were mixed.

Step	Topic and Focus Group (FG) Number						
	CINV	Fever and Neutropenia		Fertility Preservation			
	FG3	FG1	FG5	FG2	FG4	FG6	FG7
1	Education: (why) Practitioners, Pharmacists, Nursing, Buy-in	Everyone looking at the same data	Create small focus group - multi-disciplinary	Identifying AYA champion	Provider willingness and initiation to have conversation	Naming an institutional champion	Develop committee for CPG review/local adaptation
2	Defining moderately emetogenic	Tumor board (or equivalent) incorporate CPG into pre-printed order set if necessary	Review all evidence-based practices	Developing team: physicians, social worker, NP's Psychology	Identify resources needed - establish banking facility	Create task force/identify team members to have discussion with all patients	Evaluate funding/ resource availability
3	Ensure drug availability	Education (RN's, residents, families)	Define 'afebrile'. Define bone marrow recovery	Identifying a facility for sperm banking	Define patient population needing conversation and provider education	Include patient and/or family rep in planning/implementation	Designated champion to oversee implementation of process / Include APRN in initial diagnosis discussion for AYAs
4	Compatibility of drugs	RN's know exact time (Develop tools to allow RN's to track, Hard stop in EMR, Handoff - RN or Resident, Visual Cue)	Present information to stakeholders	Identifying funding	Procedure for collection	Finding FP Specialist	Develop standard process plan/logistics (labs, kits, samples pick up, etc. location, wifi / materials

Step	Topic and Focus Group (FG) Number						
	CINV	Fever and Neutropenia		Fertility Preservation			
	FG3	FG1	FG5	FG2	FG4	FG6	FG7
5	EMR template (CPOE, Insurance, Alternatives, Exceptions)	Creating guidelines for RN's (Checking results and communication - action items for RNs)	Seek approval from institutional safety board	Education: team, family, patient	Standardized Protocol Initiated - power plan for new dx --> order set EHR -> prompt in any appropriate chemo order set	Finding out cost - Family, institution	Designate team (MD/RN/SW) to provide info to patient/family
6	Patient/Family education (continuing education of users)	Multidisciplinary Rounds (MD-led education, with required attendance, empowering families, incorporating guideline into script at rounds)		Integration into care/treatment process (including logistical info)	Patient education materials - standardized packet	Create supportive docs (posters, pamphlets) for staff and families	Cultural/Patient sensitivity including privacy
7	Monitoring for compliance (Pharmacy audit, EMR query, Re-evaluating fallouts	Auditing to determine adherence (Managing non-compliers, chart requires)		Provision of emotional support	CPG Implementation audit process - feedback	Create step-by-step algorithm to provide care/FP	
8						Include prompt/link to EMR/include in standard order set	
9						Staff education	

FG: focus group; CINV: chemotherapy-induced nausea and vomiting
